# Whole genome analysis of extensively drug resistant *Mycobacterium tuberculosis* strains in Peru

**DOI:** 10.1038/s41598-021-88603-y

**Published:** 2021-05-04

**Authors:** David Santos-Lazaro, Ronnie G. Gavilan, Lely Solari, Aiko N. Vigo, Zully M. Puyen

**Affiliations:** 1 Instituto Nacional de Salud, Lima, Peru; 2 Escuela de Medicina, Universidad Peruana de Ciencias Aplicadas, Lima, Peru; 3 Escuela Profesional de Medicina Humana, Universidad Privada San Juan Bautista, Lima, Peru

**Keywords:** Comparative genomics, DNA sequencing, Next-generation sequencing, Phylogeny, Infectious-disease diagnostics, Antimicrobial resistance

## Abstract

Peru has the highest burden of multidrug-resistant tuberculosis in the Americas region. Since 1999, the annual number of extensively drug-resistant tuberculosis (XDR-TB) Peruvian cases has been increasing, becoming a public health challenge. The objective of this study was to perform genomic characterization of *Mycobacterium tuberculosis* strains obtained from Peruvian patients with XDR-TB diagnosed from 2011 to 2015 in Peru. Whole genome sequencing (WGS) was performed on 68 XDR-TB strains from different regions of Peru. 58 (85.3%) strains came from the most populated districts of Lima and Callao. Concerning the lineages, 62 (91.2%) strains belonged to the Euro-American Lineage, while the remaining 6 (8.8%) strains belonged to the East-Asian Lineage. Most strains (90%) had high-confidence resistance mutations according to pre-established WHO-confident grading system. Discordant results between microbiological and molecular methodologies were caused by mutations outside the hotspot regions analysed by commercial molecular assays (*rpoB* I491F and *inhA* S94A). Cluster analysis using a cut-off ≤ 10 SNPs revealed that only 23 (34%) strains evidenced recent transmission links. This study highlights the relevance and utility of WGS as a high-resolution approach to predict drug resistance, analyse transmission of strains between groups, and determine evolutionary patterns of circulating XDR-TB strains in the country.

## Introduction

Tuberculosis (TB) is a preventable and curable disease and one of the top 10 causes of death in the world^1^. Resistance to drugs used in the treatment of TB is a major threat to the strategies that are being deployed to control and eliminate the multidrug-resistant TB (MDR-TB) and extensively drug-resistant TB (XDR-TB)^2–4^. Both forms of this pathology are becoming more prevalent through the years and require an expensive and prolonged treatment that produces greater morbidity, toxicity and mortality^2,5^. In 2019, it was reported that 3.3% of new TB cases and 18% of previously treated cases globally were diagnosed with MDR-TB, of which an estimated proportion of 6% (12,350 reported cases) were XDR-TB^1^. Moreover, XDR-TB has the lowest treatment success rate (39%) compared to other forms of TB^2^.

In the region of the Americas, XDR-TB is present being Peru the country with the highest burden^1^. The first Peruvian case of XDR-TB was detected in 1999^6^, and throughout the years the number of cases has progressively increased, adding up to 944 cases until 2017^7,8^. The national distribution of XDR-TB corresponds to the epidemiological situation of TB in the country. Approximately, 85–88% of XDR-TB cases are concentrated in the capital city of Lima and Callao, with the eastern part of Lima being particularly one of the areas with the highest number of cases of this form of TB^7,9^.

Drug resistance of mycobacterial strains is detected through genotypic or phenotypic laboratory tests that detect the presence of DNA mutations conferring resistance or the growth of *Mycobacterium tuberculosis* (MTB) in the presence of anti-TB drugs, respectively. However, these methodologies are restricted to the analysis of a limited number of resistance genes or to the slow mycobacterial duplication time, respectively. All of this makes it difficult to obtain complete and rapid results. To determine genetic relationships, analysis of the restriction fragment length polymorphisms of the *IS6110* gene (IS6110-RFLP), spacer oligonucleotide typing (Spoligotyping) and mycobacterial interspersed repetitive units-variable number of DNA tandem repeats (MIRU-VNTR) have been used globally. These methodologies have also been applied in Peru for exploration of the genetic diversity of drug resistant TB strains^10–12^. However, the problem using these conventional techniques for genotyping is that they explore polymorphic genetic regions that cover less than 1% of the mycobacterial genome, significantly limiting their power of differentiation between strains that are very close at the genetic level^13,14^.

Recently, the revolution of Next Generation Sequencing (NGS) technology and its wider availability have allowed to perform Whole Genome Sequencing (WGS) analysis to provide information about speciation, drug resistance prediction and better determination of relatedness for epidemiologic purposes^15–17^. In this way it is possible to obtain a greater amount of information that allows a complete characterization and discrimination of strains with repeated or ambiguous conventional genotypic patterns^18,19^. However, to date no high-resolution genomic study has been performed on Peruvian XDR-TB strains.

The objective of this study is to characterize the genomic variability of the XDR-TB strains circulating in Peru, using the NGS-based WGS analysis. We performed an approximation of their molecular epidemiology and determined the evolutionary relationships of the XDR-TB Peruvians strains.

## Methods

### Sample collection

MTB strains from hospitals and public health laboratories throughout Peru are sent daily to the National Reference Laboratory for Mycobacteria (NRLM), of the Peruvian National Institute of Health (NIH), for TB confirmation and evaluation of antimicrobial susceptibility. A total of 68 XDR-TB strains, according to phenotypic results, stored at NRLM were included in this study. All recovered strains correspond to patients with active pulmonary TB diagnosed between 2011 and 2015. These strains were randomly selected from the entire country.

### Ethical statement

Approval for the use and processing of preserved strains was obtained from the Institutional Committee for Research Ethics of the Peruvian NIH (reference number OT-0021-17). Identity of the patients was blinded to the researchers using a dual coding system from this study.

### Genotypic and phenotypic confirmation

Cryopreserved MTB strains were inoculated in Middlebrook 7H9 media (Becton Dickinson, Sparks, USA) for seven days. Subsequently, 0.2 mL of 7H9 supernatant was transferred to Lowenstein–Jensen (LJ) medium and incubated for a minimum of three weeks to obtain a moderate development. Genotypic confirmations of resistance against rifampicin and isoniazid were performed using the line probe assay GenoType MTBDR*plus* v2 (Hain Lifescience, Nehren, Germany), according to the manufacturer's protocol. Phenotypic confirmation was performed using the proportion method (PM) in Middlebrook 7H10 media (Becton Dickinson, Sparks, USA) to assess the resistance to isoniazid (0.2 μg/mL, 1.0 μg/mL), rifampicin (1.0 μg/mL), levofloxacin (1.0 μg/mL), capreomycin (10.0 μg/mL) and kanamycin (5.0 μg/mL). All the work related to the manipulation of live bacteria was carried out in facilities with biosafety level 3 of the NRLM.

### DNA extraction and whole genome sequencing

Genomic DNA extractions were performed using the GeneJET Genomic DNA Purification kit (Thermo Fisher Scientific, Waltham, USA) according to manufacturer's recommendations. Double-stranded DNA concentration was quantified using the Qubit dsDNA HS Assay kit (Thermo Fisher Scientific, Waltham, USA). Sequencing libraries were prepared using 1 ng of each DNA sample with Nextera XT Library Preparation kit. Whole genome sequencing was carried out at NIH (Lima, Peru) using Illumina MiSeq platform (Illumina, San Diego, USA) to generate paired-end sequencing reads.

### Bioinformatic analysis

All computational analyses were performed by the bioinformatics department of the NRLM and were entirely set on the ubuntu distribution of Linux.

#### Purity assessment and quality filtering

Quality evaluation of paired-end reads was performed using FastQC v0.11.9 (https://www.bioinformatics.babraham.ac.uk/projects/fastqc). The presence of specific reads for *M. tuberculosis* complex species was verified with Kraken2 v2.0.7 (MiniKraken2 v2 database)^20^. Verified paired-end reads were filtered with Trimmomatic v0.38^21^ using default values and a minimum Phred score of 20. Only filtered paired-end reads (95.6% of total raw reads) were used for downstream analysis.

#### Assembly, alignment, and variant calling

Filtered paired-end reads were mapped against the H37Rv reference genome (GenBank accession number: NC_000962.3) using BWA v0.7.17^22^. Identification of duplicate reads and sorting were done with Picard-tools v2.18.25 (http://broadinstitute.github.io/picard). Mapping depth and coverage was determined using samtools v1.9^23^, bedtools v2.29.0^24^ and a custom script in R v3.6.1^25^. For variant call a local realignment of mapped reads was performed using HaplotypeCaller algorithm, implemented in GATK v3.8^26^. A hard-filtering approach was performed with VCFtools v0.1.16^27^ to select variants with the following criteria: mapping quality ≥ 60, variant depth ≥ 10X and frequency of reads supporting alternate allele ≥ 0.75. Genome positions with missing genotypes (due no coverage of reads or less than 10X depth coverage) in a minimum of 10% of all strains, and variants identified in repetitive regions (PE, PPE and PE-PGRS families) were excluded. Selected variants were annotated using SnpEff v4.3 T^28^. Concatenated genome-wide Single Nucleotide Polymorphisms (SNPs) sequences were generated for subsequent analysis.

#### Resistance genetic variants

Resistance-associated genes were analysed to evaluate phenotypic resistance to rifampicin (*rpoB*, *rpoC*, *rpoA*), isoniazid (*katG*, *inhA*, *mabA*, *kasA*, *furA*, *ndh*, *mshA*, *nat* and *oxyR-ahpC* region), levofloxacin (*gyrA, gyrB*) and second-line injectable drugs (*rrs, eis, tlyA*). Variant allelic frequency of at least 0.10 was set for these genes. Resistance genetic variants were visually confirmed using the Artemis v18.1^29^. Variants were compared with those reported in the TB Drug Resistance Mutation Database (https://tbdreamdb.ki.se) and confidence was graded based on the technical guide to resistance-associated mutations reported by the World Health Organization (WHO)^30^.

#### Lineage/sublineage and family determination

MTB lineages and sublineages determination was performed with Kvarq v0.12.2^31^ using the set of SNPs proposed by Coll *et al*.^32^. MTB families were determined using the in silico detection of 43 unique spacers in the direct repeat locus using SpoTyping v2.0^33^. Then, the presence or absence of this spacers were analysed in the SITVIT2 database (http://www.pasteur-guadeloupe.fr:8081/SITVIT2) for the determination of the corresponding ‘Spoligo-International-Type’ (SIT).

#### Evolutionary analysis

A maximum likelihood phylogenomic tree was built from concatenated genome-wide SNPs using RAxML-NG v0.9.0^34^. A 25 random and 25 parsimony-based starting trees and 1000 standard non-parametric bootstrap replicates were used to assess branch support. A general time-reversible substitution model was selected based on Akaike’s information criterion using jModelTest2^35^. The tree was rooted using a ‘Lineage seven’ strain (SRA ID: ERR181435). An alternative phylogenomic tree was built using additional non-Peruvians 221 XDR-TB strains for an international comparison (Supplementary Table S1).

#### Transmission clusters determination

Genomic transmission clusters were determined using genome-wide SNPs independently of the epidemiological data. A cut-off value of no more than 10 SNPs distance, pre-established for a high prevalence area^36–39^, was used to group the strains into the same recent transmission genetic cluster. SNP distances were obtained from nucleotide pairwise comparisons of all sequenced strains using the R package Ape v5.4^40^. Transmission network was constructed using the SeqTrack^41^ algorithm from R package Adegenet v2.1.3^42^.

## Results

### Patient characteristics

68 XDR-TB strains were included. Five (7.4%) strains were obtained from patients diagnosed in 2011, two (2.9%) in 2012, 18 (26.5%) in 2013, 23 (33.8%) in 2014 and 20 (29.4%) in 2015. Likewise, 38 (55.9%) strains were initially obtained from men and the rest from women. Their age ranged from 15 to 78 years with a mean of 36 years (interquartile range 25.3–44.5). 51 (75.0%) cases had received a previous anti-TB treatment (Table 1). 58 (85%) strains belonged to patients from Lima region and Callao province (51 from Lima and 7 from Callao). Regarding the strains of the Lima region, 30 (59%) came from districts of the east zone*,* 16 (31%) from the centre and north, 3 (6%) from the south, and 2 (4%) from other provinces (Supplementary Fig. S1). In addition, Piura, La Libertad, Loreto, Ancash, Ucayali, Arequipa and Madre de Dios regions had one strain each, while the Ica region had three strains (Supplementary Table S2).

**Table 1:** Characteristics of patients with XDR-TB.

Characteristics	n [%]
**Sex**	
Male	38 [56]
Female	30 [44]
**Treatment History**	
Previously treated	51 [75]
Newly diagnosed	15 [22]
Unknown	2 [3]
**Age**	
≤ 15 year	1 [1]
16–30 year	24 [35]
31–45 year	25 [37]
> 45 year	16 [24]
Unknown	2 [3]
Mean (IQR)	36.3 (25–45)
Resistance to SLI*	
Kanamycin resistant	11 [16]
Capreomycin resistant	14 [21]
Kanamycin & Capreomycin resistant	43 [63]
**Lineage**	
Euro-American	62 [91]
East-Asian	6 [9]

*IQR* interquartile range, *SLI* Second-Line Injectable.

*Results obtained from Proportion Method in Middlebrook 7H10 agar. All strains had additional phenotypic resistance to rifampicin, isoniazid, and levofloxacin.

### Sequencing and genome assembly

An average of 935,183 raw sequencing reads per fastq file were obtained. Two fastq files (*forward* and *reverse*) were generated for every sample. The minimum, maximum and average genome depth of sequencing obtained were 53X, 153X and 88X, respectively. All strains had reads covering more than 99% of the H37Rv genome (Supplementary Table S2).

### Antimicrobial resistance

All strains showed simultaneous phenotypic resistance to rifampicin, isoniazid, and levofloxacin. However, they showed differences in resistance to second-line injectable drugs (Table 1). Discordant results between phenotypic and genotypic methods were found for isoniazid (strain XDR-28) and rifampicin (strain XDR-19) showing resistance results only through the phenotypic method (Supplementary Table S2) and were analysed in another study^43^. Concerning rifampicin resistance, all strains had resistance mutations located at *rpoB* gene. From these, 67 (98.5%) were considered high-confident mutations located inside the rifampicin-resistance-determining region (RRDR) of the *rpoB* gene, while only one strain (1.5%) presented a mutation outside this region (I491F). The most frequent mutations were S450L and D435V. Only one strain had the double mutation H445N + S431R. The isoniazid resistant strains showed mutations in *katG* and *inhA* genes. Only one mutation in *katG* (S315T), three mutations in the promoter region (g-17t, c-15t and t-8c) and one mutation in the coding region (S94A) of *inhA* were found. There were four strains with double mutation, S315T + c-15t, and one with S315T + t-8c (Table 2). Levofloxacin resistance was predominately caused by mutations occurring in the quinolone-resistance-determining region (QRDR) of *gyrA* gene and contained nine different mutations, whereas *gyrB* showed only three. In the *gyrA* gene, the codon 94 showed the greatest variability (five different mutations). Only two strains presented mutations in both genes. Finally, one strain (1.5%) did not present mutations in either of the two genes. Resistance to kanamycin and capreomycin was driven by mutations occurring at *rrs* (a1401g, c1402t and g1484t), *tlyA* (all frame shifts) and *eis* (c-14t) genes. However, no mutations were detected in two strains for the screened genes. Strains with exclusive resistance to kanamycin only presented the *rrs* a1401g mutation, whereas two strains had no mutations in any of the three genes analysed. Exclusive resistance to capreomycin was caused by several frameshifts’ mutations occurring in *tlyA* gene. However, there were three strains with no detected mutations in any of the analysed genes. In general, 96, 85 and 90% (average 90%) of strains had high-confident mutations for resistance to rifampicin, isoniazid and both second-line drugs, respectively (Table 2). Several synonymous and nonsynonymous mutations located in additional resistant-associated genes were evidenced to be present together with the mutations described above (Supplementary Table S3).

**Table 2:** Resistance-associated mutations in Peruvian XDR-TB strains.

Drug	Gene	Mutation	Confidence grading **	n (%)
Rifampicin	*rpoB*	Q432P	High	1 (1.5)
		D435V	High	22 (32.4)
		H445N, S431R	Minimal	1 (1.5)
		H445R	High	4 (5.9)
		H445S	No data	1 (1.5)
		S450L	High	38 (55.9)
		I491F	Minimal	1 (1.5)
Isoniazid	*katG*	S315T*	High	53 (77.9)
	*inhA*	c-15t	Moderate	7 (10.3)
		g-17t	No data	2 (2.9)
		S94A	No data	1 (1.5)
	*katG* + *inhA*	S315T* + c-15t	High	4 (5.9)
		S315T* + t-8c	High	1 (1.5)
Levofloxacin	*gyrA*	G88C	High	2 (2.9)
		A90V	High	13 (19.1)
		S91P	High	2 (2.9)
		D94A	High	2 (2.9)
		D94G	High	32 (47.1)
		D94H	No data	3 (4.4)
		D94N	High	5 (7.4)
		D94Y	High	1 (1.5)
		A90V, D94H	High	1 (1.5)
		D94N, D94G	High	1 (1.5)
		D94N, D94A	High	1 (1.5)
	*gyrB*	S447F	No data	2 (2.9)
	*gyrA* + *gyrB*	G88A + T500P	No data	1 (1.5)
		D94A + E501D	High	1 (1.5)
	Mutation not detected			1 (1.5)
Kanamycin & Capreomycin	*rrs*	a1401g	High	34 (50.0)
		c1402t	High	1 (1.5)
		g1484t	High	1 (1.5)
	tlyA	S92*fs*	Generally high	2 (2.9)
		A111*fs*	Generally high	1 (1.5)
		H68fs, S92fs, Q202fs	Generally high	1 (1.5)
	*rrs, eis, tlyA*	a1401g, c-14t, R60*fs*	High	1 (1.5)
	Mutation not detected			2 (2.9)
Kanamycin	*rrs*	a1401g	High	9 (13.2)
	Mutation not detected			2 (2.9)
Capreomycin	*tlyA*	V198*fs*	Generally high	4 (5.9)
		G232D	Generally high	2 (2.9)
		L209*dup*	Generally high	1 (1.5)
		C86*fs*	Generally high	1 (1.5)
		S252*fs*	Generally high	1 (1.5)
		R133fs, L209dup	Generally high	1 (1.5)
		L139fs, V198fs	Generally high	1 (1.5)
	Mutation not detected			3 (4.4)

Codon numbering systems are according H37Rv genome (NC_000962.3).

*fs* frame shift, *dup* duplication.

*AGC → ACC.

**According to WHO-NGS Technical guide^30^. The percentages are relative to the total of resistant strains for each drug.

### Lineages and evolutionary relationships

**Figure 1:**
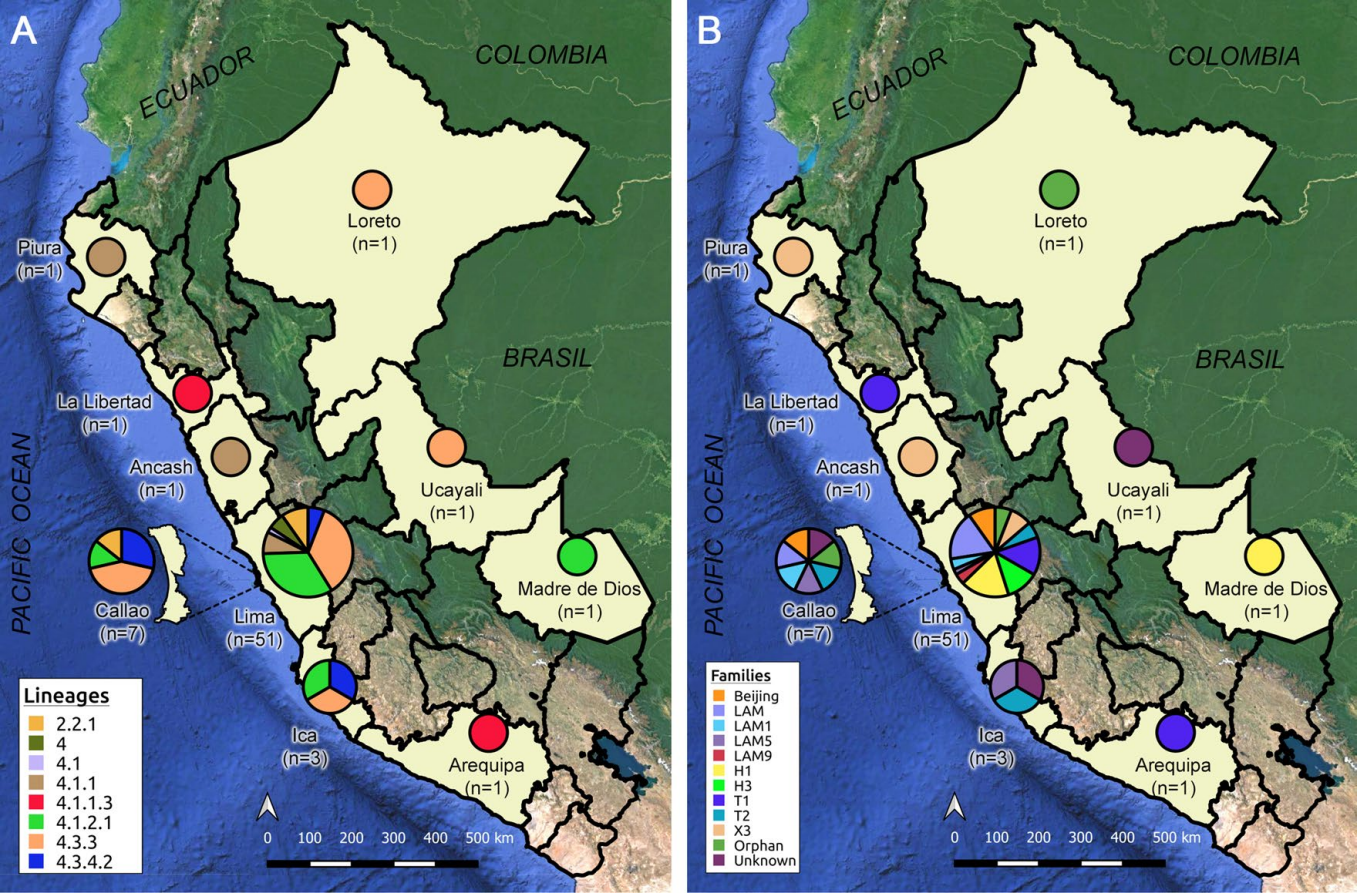
Lineages and family classification of Peruvian XDR-TB strains. Distribution of lineages, sublineages (**A**) and genetic families (**B**) throughout the entire Peruvian territory. The number of XDR-TB strains (n) from each region is specified. The size of the circles was set using the scale ‘Log_10_ (n + 1)’. Maps were created using QGIS v3.14.15 (https://www.qgis.org).

Lineage’s analysis determined that 62 (91%) XDR-TB strains belong to the Euro-American Lineage (Lineage 4) and 6 (9%) to the East-Asian Lineage (Lineage 2). 59 strains from Lineage 4 were able to be classified into six sublineages, while three could only be assigned as belonging to lineage 4. All strains from Lineage 2 were represented by sublineage 2.2.1. Five strains of Lineage 2 circulated in the Lima region, while the remaining strain circulated in the province of Callao (Fig. 1).

The presence of 18 strains belonging to the LAM family was found (LAM/SIT1355 [n = 10; 14.7%], LAM1/SIT469 [n = 3; 4.4%], LAM5/SIT93 [n = 2; 2.9%], LAM5/SIT1160 [n = 1; 1.5%], LAM9/SIT42 [n = 2; 2.9%]), 16 strains belonging to the Haarlem family (H1/SIT47 [n = 9; 13.2%], H1/SIT62 [n = 1; 1.5%], H3/SIT3001 [n = 6; 8.8%]), 14 strains belonging to the T family (T1/SIT53 [n = 6; 8.8%], T1/SIT219 [n = 2; 2.9%], T1/SIT535 [n = 1; 1.5%], T2/SIT52 [n = 5; 7.4%]), 6 strains belonging to the X family (X3/SIT91 [n = 5; 7.4%], X3/SIT3780 [n = 1; 1.5%]), and 6 (8.8%) strains belonging to the Beijing/SIT1 family. Likewise, the presence of a reduced number of strains with orphan (n = 4; 4%) and unknown (n = 4; 4%) spoligotypes was also observed (Supplementary Fig. S2 and Table S2).

The northern strains of the country belonged to Piura (4.1.1/X3/SIT91; n = 1), La Libertad (4.1.1.3/T1/SIT219; n = 1) and Loreto (4.3.3/Orphan; n = 1). In the centre of the country, the regions of Lima, Ica and the province of Callao showed to contain the greatest genetic diversity with respect to the entire country, with six, three and four sublineages respectively. The rest of the central regions were integrated by Ancash (4.1.1/X3/SIT91; n = 1) and Ucayali (4.3.3/Unknown/222; n = 1). Finally, in the South of the country, strains were found to belong to Arequipa (4.1.1.3/T1/SIT219; n = 1) and Madre de Dios (4.1.2.1/H1/SIT62; n = 1) (Fig. 1 and Supplementary Table S2).

**Figure 2:**
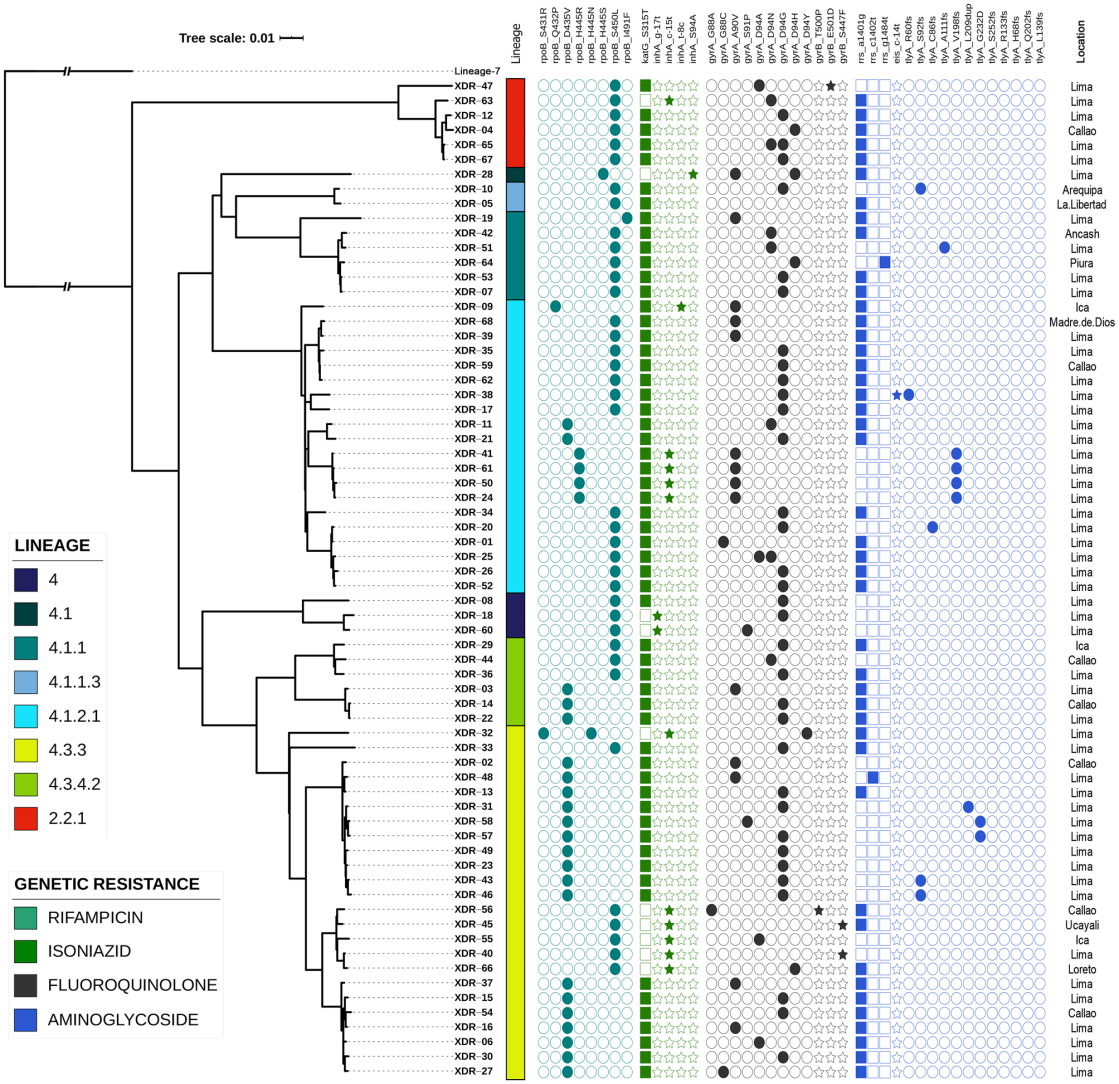
Maximum likelihood phylogenomic tree from 68 genomes of Peruvian XDR-TB strains. The tree was based on 6,076 genome-wide SNPs. More than 74% of nodes presented a support bootstrap ≥ 70%. All nodes outlining the main subdivisions of lineages and sublineages were supported by 100% bootstrap values. Lineages are represented near the tip names. Mutations are represented by filled (presence of mutation) or empty (absence of mutation) icons. The figure was created using iTOL v5 (https://itol.embl.de).

The maximum likelihood phylogenomic tree confirmed the lineage and sublineage classification and showed additional subclassification for strains belonging to the same sublineage. Interestingly, no correlation between phylogenomic clades and specific mutations conferring resistance was observed. Spoligotypes with ‘Orphans’ and ‘Unknown’ SITs could be characterized by evolutionary and lineage analysis. Three strains with ‘Unknown’ SITs got aligned within the sublineage 4.3.3 group, showing an evolutionary similarity with members of the H3 family, while the strains with ‘Orphans’ SITs were in the groups belonging to sublineages 4.3.3 (n = 3) and 4.1.2.1 (n = 1) (Supplementary Fig. S3). A close evolutionary relationship was found between strains from Arequipa (XDR-10) and La Libertad (XDR-05), being the only representatives of the sublineage 4.1.1.3 in the entire country and exhibiting an important degree of genomic differentiation with the other strains (Fig. 2). Global evolutionary relatedness showed that Peruvian XDR-TB strains were grouped in different monophyletic clades and had a close relatedness with XDR-TB strains of Lineages 4 and 2 of European countries (Supplementary Fig. S4).

### Transmission cluster determination

**Figure 3:**
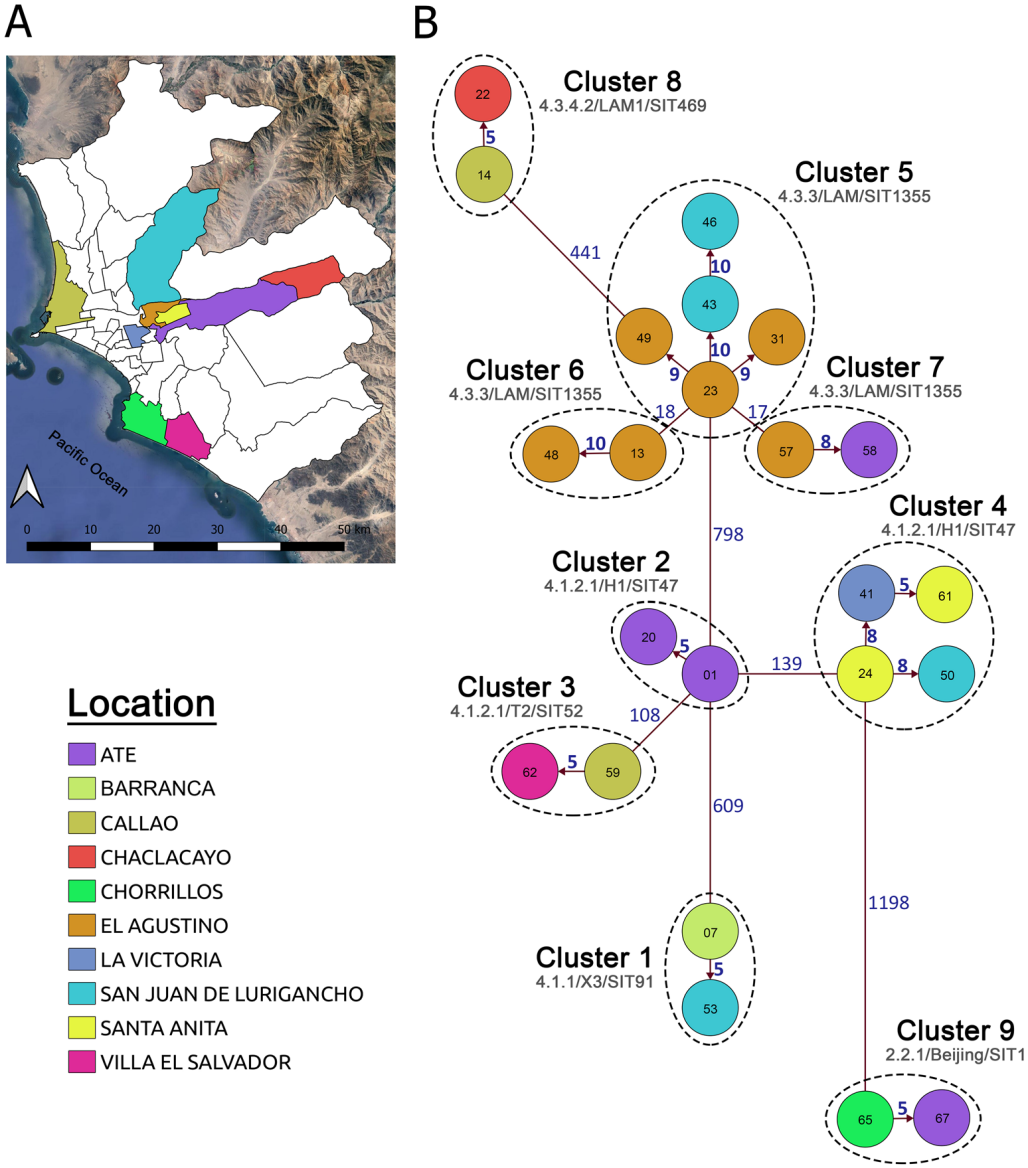
Peruvian XDR-TB strains cluster analysis. (**A**) Map of the districts of Lima and Callao that contain XDR-TB strains associated in transmission clusters. Barranca is not specified since it is located outside Lima (north side). (**B**) Inferred transmission network based on pairwise genetic distances. Each node represents one XDR-TB strain. The number of SNPs that separate the different strains within and between clusters is specified. Arrows indicate the potential direction of transmission within clusters. The node numbers are derived from the ‘XDR-#’ format in Fig. 2. Figure 3A was generated with QGIS v3.14.15 (https://www.qgis.org). Figure 3B was generated using R package Igraph v1.2.6 (https://igraph.org/r).

The analysis of pairwise genetic differences showed a high number of strains that differed by large amounts of SNPs. The genetic distance between strains varied from 5 to 1,272 SNPs with an interquartile range of 373 and a median of 772 (Supplementary Fig. S5). The WGS analysis determined that most strains were not related at genetic level with only 23 strains forming part of nine transmission clusters (clustering rate of 34%), each one comprising between two and five strains (Fig. 3 and Supplementary Fig. S6). These clusters consisted mainly of strains from Lineage 4 (91.3%). The largest clusters were integrated by strains of the sublineages 4.3.3 (cluster 5; n = 5) and 4.1.2.1 (cluster 4; n = 4). Clusters 1 (sublineage 4.1.1/X3/SIT91) and 8 (sublineage 4.3.4.2/LAM1/SIT469) were integrated by strains with sublineages not present in the other clusters and presented an average distance to the closest cluster of 525 SNPs. The rest of the clusters of Lineage 4 were integrated by strains of sublineages 4.1.2.1 and 4.3.3. Thus, clusters 2 and 4 (sublineage 4.1.2.1/H1/SIT47), and 3 (sublineage 4.1.2.1/T2/SIT52) presented an average distance between them of 123.5 SNPs. While clusters 5, 6 and 7 (sublineage 4.3.3/LAM/SIT1355) presented an average distance between them of 17.5 SNPs. The cluster belonging to Lineage 2 (sublineage 2.2.1/Beijing/SIT1) had a considerable minimum distance of 1198 SNPs with the nearest cluster of Lineage 4 (Fig. 3).

All clustered strains came from Lima region (n = 21) and Callao province (n = 2). Regarding the strains of Lima region, 17 belonged to the east zone, one to the centre (in the area bordering the east zone), two to the south zone and one to the provinces (Supplementary Table S4). Regarding the variability of sources of infection, clusters 2 and 6 were composed of strains belonging to a single infection district (Ate and El Agustino, respectively), while the rest of clusters were integrated by at least two districts. The cluster with more members (cluster 5) was integrated by strains from the bordering districts of San Juan de Lurigancho and El Agustino. In the same way, cluster 4 was formed by four strains from the geographically close districts of San Juan de Lurigancho, Santa Anita and La Victoria. The remaining groups consisted of only two strains that came mainly from geographically separate districts (Fig. 3).

Regarding drug resistance, all the strains that made up the same cluster shared the mutations associated with resistance for rifampicin and isoniazid drugs. The strains integrating clusters 1, 2, 3, and 9 (n = 8) shared the *rpoB* S450L and *katG* S315T mutations, likewise the strains integrating clusters 5, 6, 7 and 8 (n = 11) shared the *rpoB* D435V and *katG* S315T mutations. Finally, the strains in cluster 4 shared the *rpoB* H445R, *katG* S315T and *inhA* c-15t mutations. Regarding resistance to second-line drugs, it was evidenced that not all the strains integrating a single cluster shared the same type of mutations. All the strains integrating clusters 1, 3 and 8 (n = 6) shared the *rrs* a1401g and *gyrA* D94G mutations. Similarly, all the strains in cluster 4 had the *tlyA* V198*fs* and *gyrA* A90V mutations. However, clusters 7 and 9 (n = 4) presented variations in resistance mutations only to levofloxacin, cluster 5 (n = 5) presented variations in resistance only to second-line injectables, and clusters 2 and 6 (n = 4) presented variations for both types of drugs (Supplementary Table S4).

## Discussion

In our study, we found that most XDR-TB strains belonged to the Euro-American lineage (Linage 4), in agreement with studies that claim that this lineage is the largest in the world and the more prevalent in America continent. In general, the geographical source of the sequenced samples was representative of XDR-TB affecting the entire country: 85% of the included strains were from Lima and Callao, virtually the same proportion reported by Alarcon^7^ and Soto^9^. Another important finding is that there is a high genomic diversity in our XDR-TB strains, based on the large number of sublineages obtained. However, recent transmission clusters were detected only in 34% of the strains analysed.

Concerning the mutations conferring resistance to antituberculosis drugs, currently in Peru, molecular resistance screening for first and second-line antituberculosis drugs is performed by the line probe assays: GenoType MTBDR*plus* v2 and GenoType MTBDR*sl* v2. However, these assays only concentrate the analysis on genetic hotspots. Consistent with previous studies, our results indicate that rifampicin resistance is mainly caused by mutations at codons 450, 445 and 435 of *rpoB* gene^44–46^. We also detected Q432P mutation which has a high confidence grade for rifampicin resistance development and S431R mutation which in turn has insufficient data according to WHO-NGS Technical Guide, despite having been associated with rifampicin resistant phenotypes^47^. The rifampicin-discordant strain presented the I491F mutation located outside the RRDR. This mutation was previously reported in Peru^43^ and WHO consider it as a variant with minimum confidence grade^30^. The isoniazid resistance was predominantly driven by the *katG* S315T (AGC → ACC) mutation followed by *inhA* c-15t. The rare variant *inhA* g-17t was also present in a low frequency in concordance to previous studies^16,48^. This mutation is indirectly detected by the GenoType MTBDR*plus* v2. Finally, the isoniazid-discordant strain presented the *inhA* S94A mutation that is known to confer isoniazid resistance in clinical and experimental studies^49^, and was also previously identified in Peruvian strains^43^. Levofloxacin resistance was mainly driven by mutations occurring at *gyrA* gene. However, it also was detected the *gyrB* E501D mutation which was previously associated with conferring resistance only to Moxifloxacin or ciprofloxacin^50,51^, although our results indicate that it also confers resistance to levofloxacin and has never been reported before in Peruvian strains. Strains that did not harbour mutations in either *gyrA* or *gyrB* genes suggests the existence of alternatives mechanisms of resistance like alterations in genes related to efflux pumps as well as DNA mimicry^52–54^. The presence of strains showing mutations in both genes was evidenced. A double mutation has previously associated with higher minimum inhibitory concentrations and may be associated with a decreased fitness^55^, but it could not be determined in this study. All only kanamycin resistant strains exhibited the *rrs* a1401g mutation which is strongly associated with resistance to high concentrations of kanamycin and amikacin^30^. Similarly, the simultaneous resistance to kanamycin and capreomycin is associated with high confidence mutations in the *rrs* gene (a1401g, c1402t and g1484t) associated with cross resistance to both drugs^30^. However, strains with no mutation at *rrs*, *eis* or *tlyA* genes suggest the presence of additional resistance mechanism like alterations in L10 and L12 genes (for capreomycin resistance)^56^ and overexpression of *whiB7* and efflux pump genes (for kanamycin resistance)^57,58^. This behaviour has been previously reported in other studies, including Peru^59,60^.

The analysis of phylogenetic SNPs and spoligotypes evidenced a predominance of Lineage 4 between the XDR-TB strains analysed, which is in accordance with studies that claim that this lineage is the largest in the world and the more prevalent in America continent. The high circulation of XDR-TB strains belonging to the sublineages 4.3.3/LAM, 4.3.3/LAM9, 4.3.4.2/LAM1, 4.3.4.2/LAM5 (n = 18) and 4.1.2.1/Haarlem (n = 10) are in accordance with the fact that they are considered the most widely distributed sublineages worldwide. Likewise, the presence of the 4.1.1/X3 sublineage was evidenced, which has been observed mainly in America^61^. The high number of strains belonging to Lineage 4 is a characteristic of America, and can be understood due to the colonization of the American continent by European emigrants (*founder effect*), which is estimated to have occurred approximately between the years 1466 and 1593^62^. The low frequency of Peruvian XDR-TB strains belonging to Lineage 2 reveals the recent incorporation of this Lineage into the territory. The same behaviour has been reported in countries such as Ecuador and Chile^63,64^. The proportion of strains belonging to the Beijing family (8.8%) is very similar to what was obtained in previous studies carried out in Peru in the years 2012 (9.3%)^65^ and in 2014 (9.2%)^12^. This permanence of the proportion of Lineage 2 strains through the years suggests that in Peru the XDR-TB strains of the Beijing family would not necessarily be associated with greater virulent capacity or drug-resistant factors as previously determined^66^. Also, our study suggests that until 2015, the XDR-TB strains belonging to Lineage 2 could still be geographically restricted to Lima region and Callao province.

In general, most sublineages, in addition to being present in some regions, were also present in Lima and Callao. This evidence the still existing centralization of the country's political-economic power in Lima and Callao. However, additionally, the appearance of local sublineages was observed such as 4.1.1.3/T1/SIT219 which was present only in the regions of Arequipa and La Libertad, 2.2.1/Beijing/SIT1 present only in the Lima region and the Callao province, and 4.1.2.1/H1/SIT62 present only in Madre de Dios. These sublineages of local distribution would suggest the existence of restricted transmission clusters present in these areas, which would include strains with different drug-resistant profiles. Coincidentally, the strains present in the geographically distant regions of La Libertad (northern Peru) and Arequipa (southern Peru) revealed a transmission nexus not so distant between them (only 28 SNPs apart) evidencing the existence of a common genetic ancestor for both regions.

Our results establish that the LAM family was dominant among the Peruvian XDR-TB strains analysed, which is consistent with that evidenced in neighbouring South American countries^67–72^. However, it disagrees with a previous study also carried out on Peruvian XDR-TB strains in which it was established that the dominant family was Haarlem^12^. One possible reason for this disagreement is that the previous study included strains from previous years (2007–2009) which suggests the possible occurrence of a shift in the prevalence of XDR-TB strain families in Peru over the years. On the other hand, a more recent study carried out on Peruvian MDR-TB strains agrees with our results, establishing the LAM family as the predominant family in Peru^73^.

The transmission clusters obtained confirm that XDR-TB strains are concentrated on the districts of Lima region which are more associated with poverty, overcrowding and less access to health systems. These demographic variables have already been previously well characterized in studies of Peru and the world, but is not being tackled appropriately^74^. The uniform composition of mutations associated with resistance to rifampicin and isoniazid drugs in the clusters establish that the transmission of strains in the same cluster possibly occurred initially at the level of MDR-TB strains. However, a deep insight into the clusters revealed a heterogeneous composition within each cluster regarding mutations associated with resistance to second-line drugs. This suggests the de novo emergence of the mutations that led to the XDR-TB phenotype. This discontinuity in the clusters of mutations associated with second-line resistance and the low clustering rate (34%) of strains grouped in recent transmission chains suggest that the main mechanism of acquiring an XDR-TB strain is not through by direct contact, but by failures in the individual treatment of less severe forms of tuberculosis. Furthermore, the large genetic distances observed indicate that XDR-TB strains are related by common remote ancestors, rather than caused by recent transmission events. However, it should be noted that the lack of genomic characterization of all XDR-TB strains circulating in the country, as well as strains isolated from relatives or direct contacts, may lead to the existence of missing links that underestimate the true proportion of recent transmission events in Peruvian community.

The fact that clusters 2, 3 and 4 shared the 4.1.2.1 sublineage (average distance of 123.5 SNPs) and that clusters 5, 6 and 7 shared the 4.3.3/LAM sublineage (average distance of 17.5 SNPs) establishes that Whole genome sequencing showed a higher resolution capacity in Peruvian XDR-TB strains compared to the classic spoligotyping system and the previously proposed barcode system^32^. However, it is important to have epidemiological information on patients that complements the genomic links, since it is possible that cases with well-established epidemiological links may escape the pre-established cut-off points^75^.

The study highlights the use of whole genome sequencing in the analysis of XDR-TB strains circulating in Peru. It is shown that the information obtained allows a high-resolution characterization of severe drug-resistant forms of TB. The use of genomic information would allow a complete characterization of drug-resistant mutations affecting MTB strains, as well as elucidate transmission links in high-prevalence communities. Furthermore, the incorporation of this methodology in the routine diagnosis of tuberculosis at the national level would improve the control of tuberculosis and its various drug-resistant forms.

## Conclusions

This study highlights the relevance and utility of performing Whole Genome Sequencing as a high-resolution approach to perform genetic analysis of XDR-TB strains circulating in Peru. We performed phylogenomic analysis based on both SNPs and spoligotypes evidencing the predominance of Lineage 4 through XDR-TB strains circulating in Peru. Also, transmission analysis indicates that the main mechanism of acquisition of XDR-TB is through failures in the individual treatment of less severe forms of tuberculosis. Finally, the prediction of resistance, determination of transmission groups, and evolutionary analysis can be effectively evaluated using WGS to improve the understanding of XDR-TB dynamics in these settings and provide precise information to improve control measures of TB in Peru.

## Study limitations

The study had the main limitation that only basic epidemiological tracing data were available for all patients whose isolates were included in the study. Likewise, we were not able to sequence all the XDR-TB strains obtained between the years 2011–2015. We only included viable strains recovered from the Peruvian NIH collection. Furthermore, due to the size of the sequencing reads obtained, the reliable genotype of the repetitive regions could not be obtained. This limited the analyses to only SNP-like variants that were not present in these regions. Finally, insertions and deletions (INDELs) were not included in the transmission analyses. It is possible that the omission of these genetic regions and INDEL-like variants could have masked additional genetic diversity in the samples evaluated.

## Supplementary Information

Supplementary Information 1.

Supplementary Table.

## Data Availability

All data generated and analysed in the study are included in this article and its supplementary files. Sequencing reads have been submitted to the National Center for Biotechnology Information (NCBI) Sequence Read Archive (SRA) under BioProject ID: PRJNA707145.
