# Supplementary material for: Whole genome analysis of extensively drug resistant Mycobacterium tuberculosis strains in Peru
Source: Sci Rep. 2021 May 4;11:9493. doi: 10.1038/s41598-021-88603-y (PMC8097007; doi:10.1038/s41598-021-88603-y)
Supplement: Supplementary file 1 — Supplementary Information 1. [file 41598_2021_88603_MOESM1_ESM.docx]

# TITLE: Whole genome analysis of Extensively Drug Resistant *Mycobacterium tuberculosis* strains in Peru

**Authors**:

- David Santos-Lazaro ([edavid.sant@gmail.com](mailto:edavid.sant@gmail.com)) **^1^**
- Ronnie G. Gavilan ([ronniegavilan@gmail.com](mailto:ronniegavilan@gmail.com)) **^1,3^**
- Lely Solari ([lelysol@hotmail.com](mailto:lelysol@hotmail.com)) **^1^**
- Aiko N. Vigo ([aiko.vt.anvt@gmail.com](mailto:aiko.vt.anvt@gmail.com)) **^1^**
- Zully M. Puyen ([zpuyeng@gmail.com](mailto:zpuyeng@gmail.com)) **^1,2^**^,^*****

***** Corresponding author

**Affiliations:**

**^1^** Instituto Nacional de Salud, Lima, Peru.

**^2^** Escuela de Medicina, Universidad Peruana de Ciencias Aplicadas, Lima, Peru.

**^3^** Escuela Profesional de Medicina Humana, Universidad Privada San Juan Bautista, Lima, Peru.

**
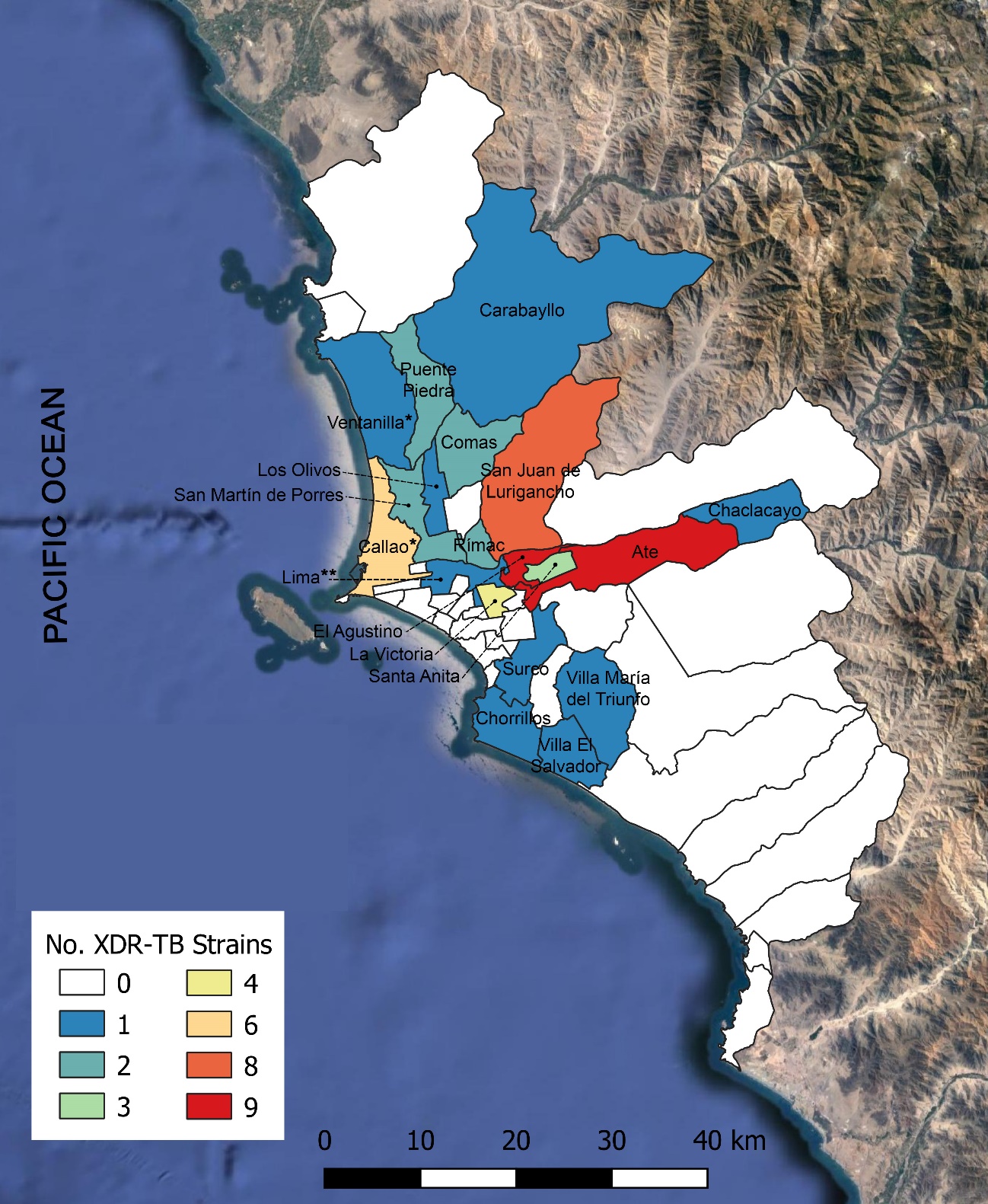
**

**Supplementary Figure S1:** Districts with XDR-TB strains in Lima region and Callao province. All XDR-TB strains from these places were distributed in 19 districts (shown in the figure) and 2 additional provinces (not shown). * Districts of the Callao province. ** Lima district. Map generated with QGIS v3.14.15 (http://www.qgis.org).


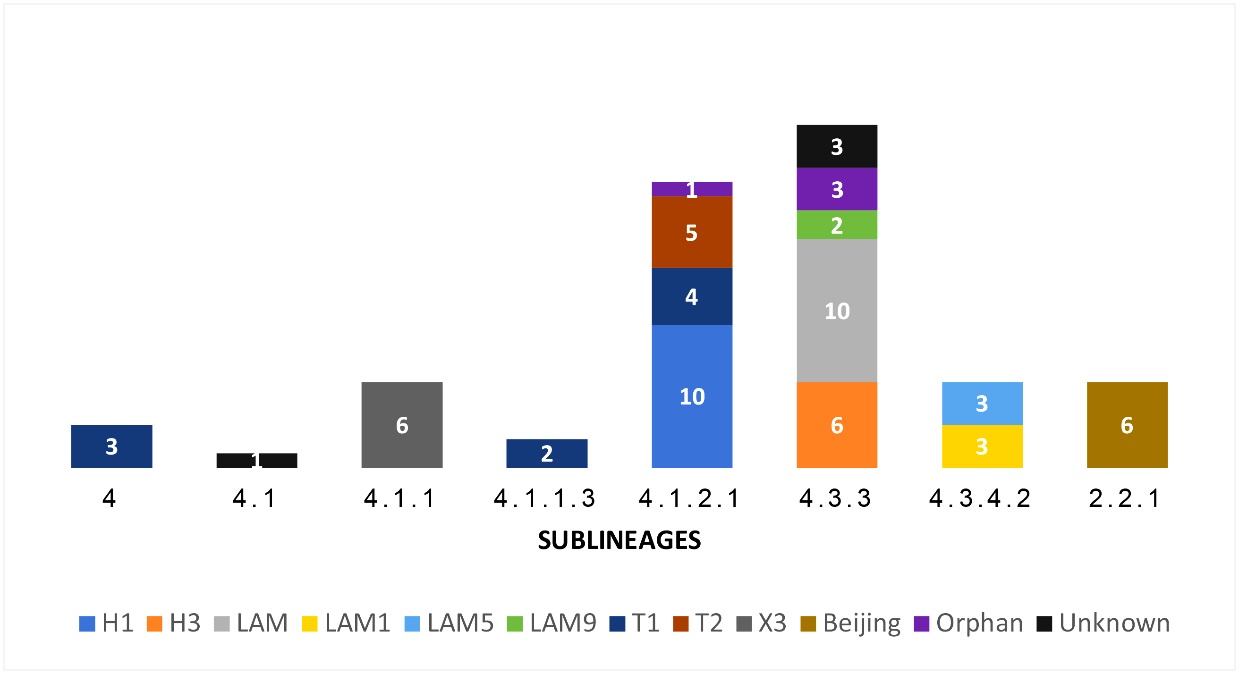


**Supplementary Figure S2:** Distribution of genetic families in the MTB lineages of the Peruvian XDR-TB strains. The number of strains of each family is specified inside the bars. Figure created with Excel v2019 (https://www.microsoft.com).

**
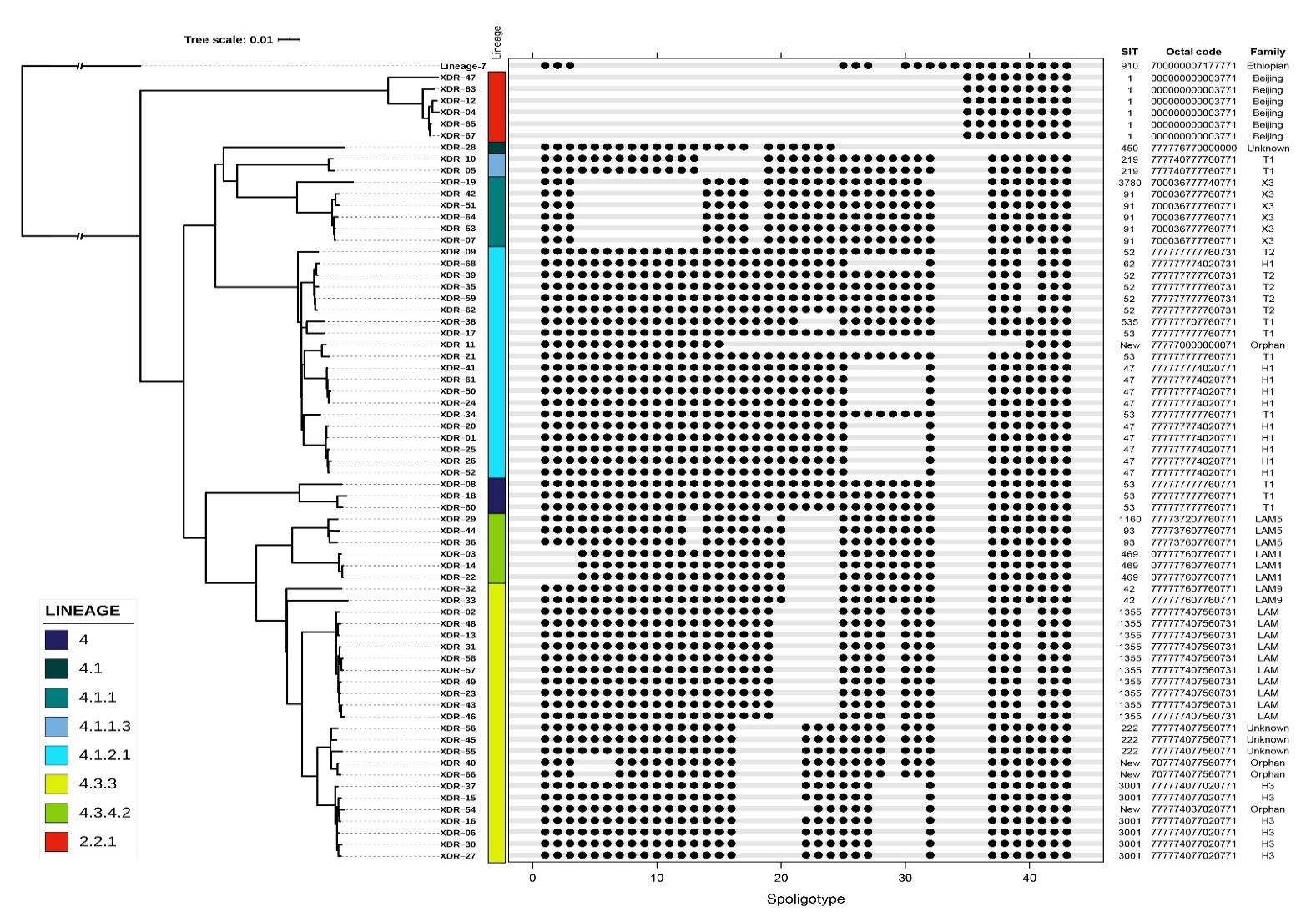
**

**Supplementary Figure S3:** *In silico* spoligotypes patterns (binary and octal codes) of Peruvian XDR-TB strains. SITs and families were annotated using the SITVIT2 database. The patterns are correlated with Lineages and the Maximum likelihood tree previously generated. **SIT**: spoligotype international type. The phylogenomic tree is the same one generated in Figure 2. Binary spoligotype patterns were generated with R package ggplot2 v3.3.2 (https://ggplot2.tidyverse.org).

**
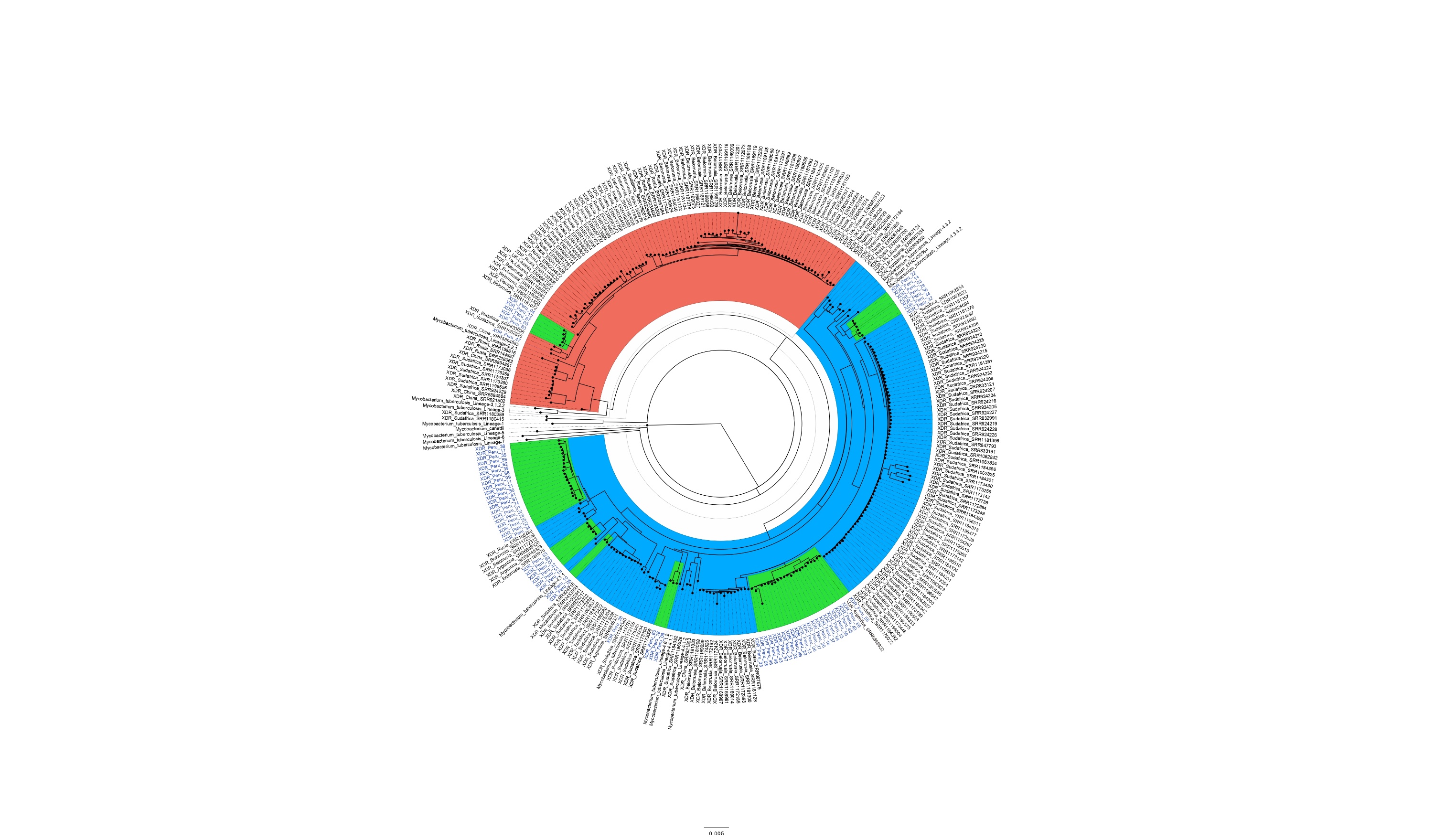
**

**Supplementary Figure S4:** Global phylogenomic tree of 289 XDR-TB strains belonging to South America, Europe, Africa and Asia. The tree was constructed using 16,798 genome-wide SNPs**.** Clusters containing Peruvian strains are highlighted in green. The tree was rooted at *Mycobacterium canettii* strain (GenBank, Access number: NC_019950.1). The figure was created using FigTree v1.4.4 (https://github.com/rambaut/figtree).


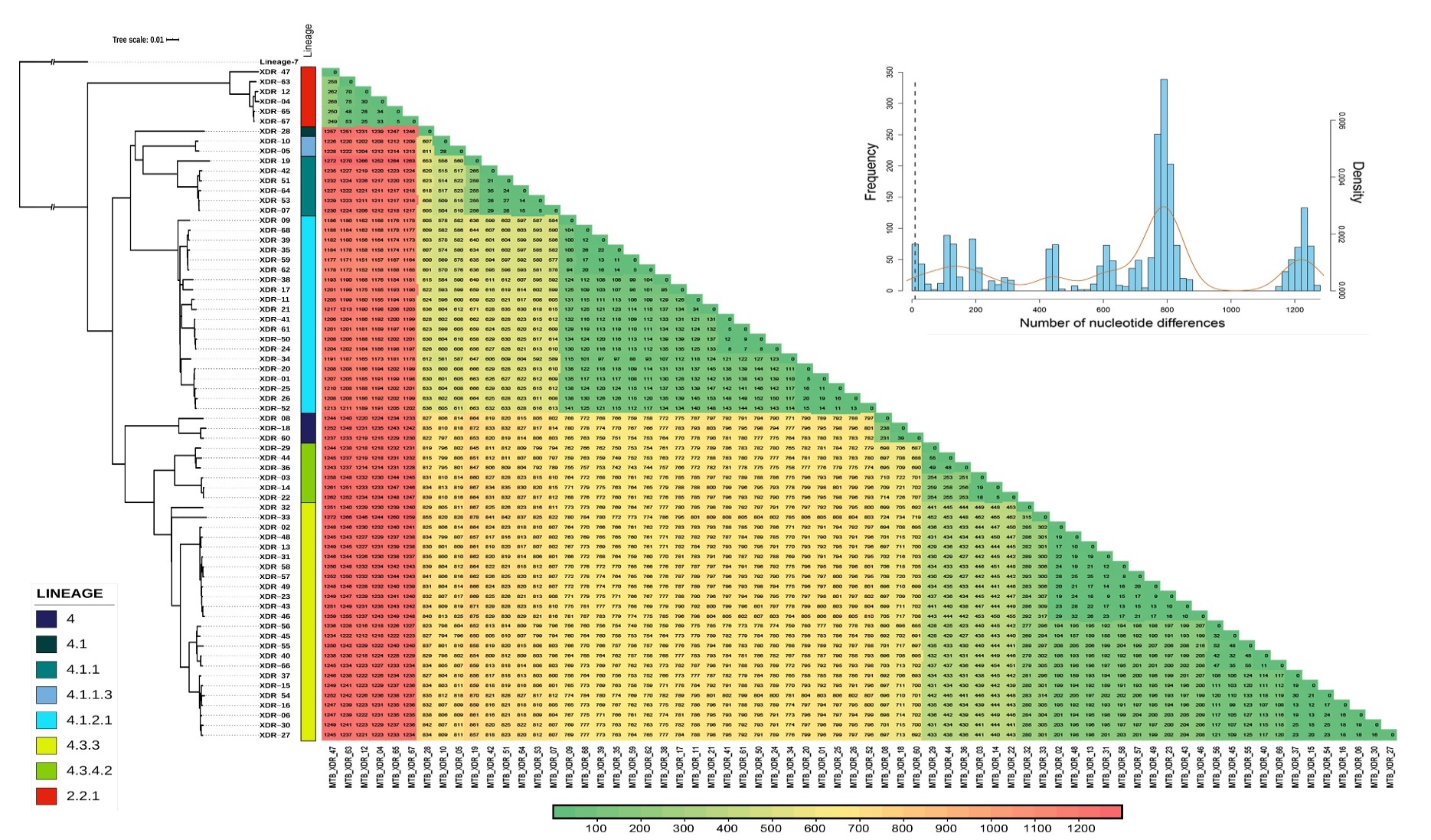


**Supplementary Figure S5:** Correlation between phylogenomic tree and nucleotide distance matrix of all XDR-TB strains. At top right is illustrated the histogram of pairwise distance (Hamming distance) and vertical dotted line indicates a cut point of 10 nucleotides differences. The phylogenomic tree is the same one generated in Figure 2. Distance matrix was created with R package Ape v5.4 (http://ape-package.ird.fr).

**
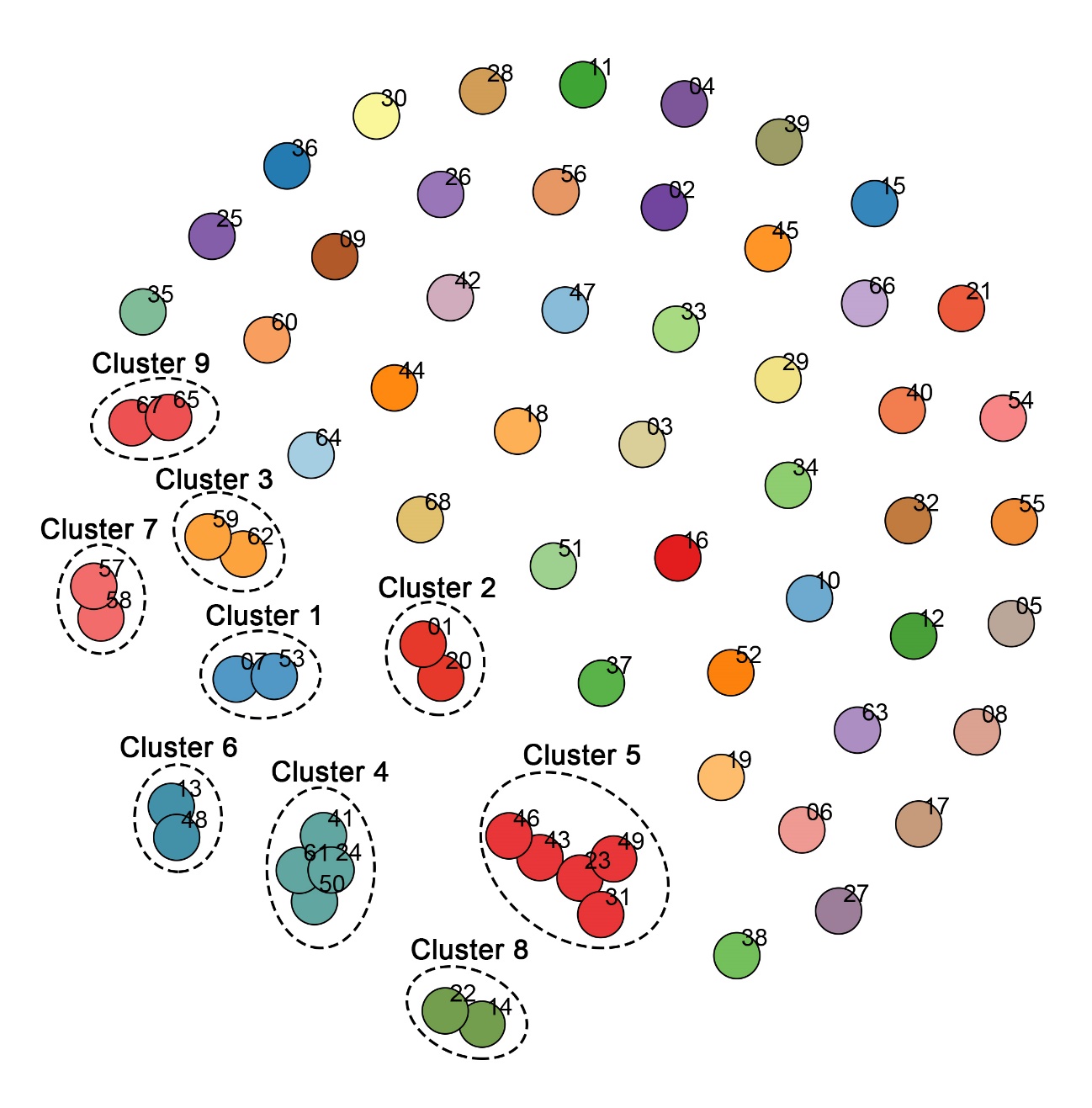
**

**Supplementary Figure S6:** Cluster determination of all XDR-TB strains. Potential recent transmission links were detected using a cut-off ≤ 10 SNPs. Strain’s numbering is according ‘XDR-***#***’ format used in the rest of figures (only the ‘***#***’ value is showed in the upper right of each node). The figure was generated with R package Adegenet v2.1.3 (https://github.com/thibautjombart/adegenet).
